# Predictive Value of the Age, Creatinine, and Ejection Fraction (ACEF) Score in Cardiovascular Disease among Middle-Aged Population

**DOI:** 10.3390/jcm11226609

**Published:** 2022-11-08

**Authors:** Shengjun Xiong, Shizhang Yin, Wanshu Deng, Yuanhui Zhao, Wenhang Li, Pengbo Wang, Zhao Li, Hongmei Yang, Ying Zhou, Shasha Yu, Xiaofan Guo, Yingxian Sun

**Affiliations:** Department of Cardiology, The First Hospital of China Medical University, 155 Nanjing North Street, Heping District, Shenyang 110001, China

**Keywords:** ACEF score, cardiovascular disease, CHD, stroke

## Abstract

Purpose: To explore the predictive value of ACEF scores for identifying the risk of cardiovascular disease (CVD) in the general population. Methods: A total of 8613 participants without a history of CVD were enrolled in the follow-up. The endpoint was CVD incidence, defined as stroke or coronary heart disease (CHD) diagnosed during the follow-up period. Cox regression analyses were used to calculate hazard ratios (HRs) with respect to the age, creatinine, and ejection fraction (ACEF) scores and CVD. A Kaplan–Meier curve was used to analyze the probability of CVD in different quartiles of ACEF. Restricted cubic spline was used to further explore whether the relationship between ACEF and CVD was linear. Finally, we assessed the discriminatory ability of ACEF for CVD using C-statistics, net reclassification index, and integrated discrimination improvement (IDI). Results: During a median follow-up period of 4.66 years, 388 participants were diagnosed with CVD. The Kaplan–Meier curve showed that ACEF was associated with CVD, and participants with high ACEF scores were significantly more likely to be diagnosed with CVD compared to participants with low ACEF scores in the general population. In the multivariate Cox regression analysis, the adjusted HRs for four quartiles of ACEF were as follows: the first quartile was used as a reference; the second quartile: HR = 2.33; the third quartile: HR = 4.81; the fourth quartile: HR = 8.00. Moreover, after adding ACEF to the original risk prediction model, we observed that new models had higher C-statistic values of CVD than the traditional model. Furthermore, the results of both NRI and IDI were positive, indicating that ACEF enhanced the prediction of CVD. Conclusions: Our study showed that the ACEF score was associated with CVD in the general population in northeastern China. Furthermore, ACEF could be a new tool for identifying patients at high risk of primary CVD in the general population.

## 1. Introduction

Cardiovascular diseases (CVDs) remain a major burden on global healthcare. In recent years, the prevalence of cardiovascular diseases has increased significantly in China [1,2]. With social development and lifestyle changes, the epidemiology of cardiovascular diseases has shifted, and regional differences in CVDs are becoming more apparent [3,4]. To develop effective and timely strategies for addressing the challenges of the CVD epidemic, we require accurate and early risk stratification.

Several models and scores that can identify CVDs in certain populations are available [5,6,7]. The age, creatinine, and ejection fraction (ACEF) score is a simple cardiovascular risk score that has garnered significant interest for its accuracy and clinical applicability [8,9,10]. The ACEF score is a good predictor of cardiovascular events in patients with a variety of diseases. However, no studies have explored the predictive value of ACEF scores for CVD risk in the general population.

This study aimed to verify the predictive value of the ACEF score for CVD and evaluate whether the ACEF score is an effective tool for cardiovascular risk stratification in the general population.

## 2. Method

### 2.1. Study Population and Ethics

**Figure 1:**
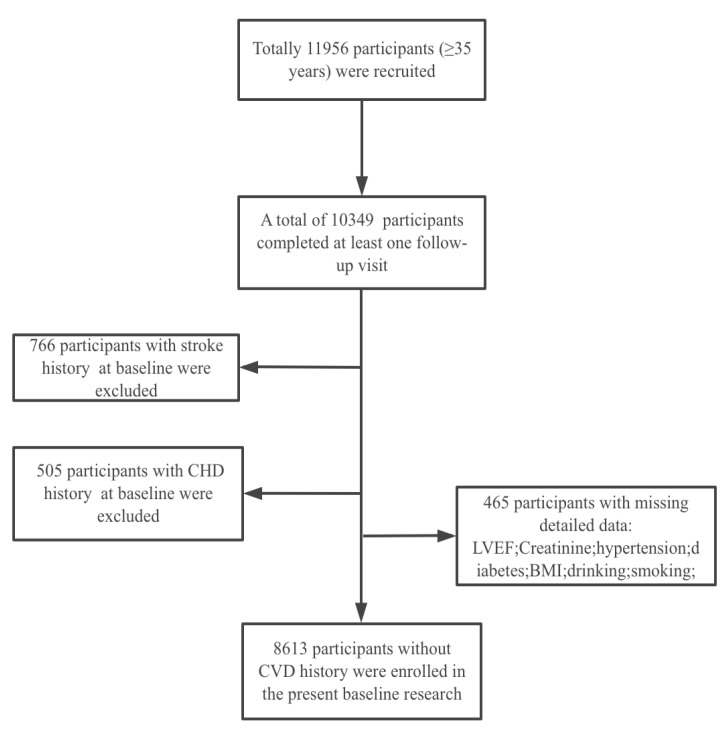
Flowchart illustrating the study population. A total of 10,349 participants were collected. A total of 8613 participants without CVD history were enrolled.

Participants included in our study were selected from the Northeast China Rural Cardiovascular Health Study, a rural community-based prospective cohort study. The design and inclusion criteria of the study have been described previously [11,12]. Participants whose age was less than 35 years or those who were pregnant, diagnosed with any mental health conditions, or had failed to complete the relevant assessments were excluded. As shown in the flow chart (Figure 1), a total of 11,956 participants aged ≥35 years from Dawa, Zhangwu, and Liaoyang counties in Liaoning province were enrolled. A number of 10,349 participants completed at least one follow-up visit. A total of 766 and 505 participants with stroke history and coronary heart disease (CHD) history, respectively, at baseline were excluded. Furthermore, 465 participants with missing data on corresponding factors such as LVEF, creatinine, hypertension, diabetes, body mass index (BMI), alcohol consumption, and smoking status were excluded. These participants with missing relevant information were tested, and the corresponding data were not statistically different from those of the study population at baseline. Finally, data from 8613 participants without CVD history were available for analysis. The study was approved by the Ethics Committee of China Medical University. All procedures were performed in accordance with ethical standards. After preliminary screening, subjects provided their information and written consent to participate in our study. All data collection, storage, and analysis procedures were performed in accordance with approved ethical protocols. If the participants were illiterate, written informed consent was obtained from their proxies.

### 2.2. Data Collection

Cardiologists collected data during clinical visits, and trained nurses used a standard questionnaire via face-to-face questioning. Before conducting the survey, eligible investigators attended training, and those who passed the training test were allowed to participate in our study. Our questionnaire collected data, including age, gender, smoking status, alcohol consumption, and personal history. Blood pressure was collected by two trained nurses using an automatic electronic sphygmomanometer (HEM-907; Omron, Kyoto, Japan). Blood pressures were measured thrice, and the average was then calculated. We asked the subjects to remove their shoes before evaluating a range of anthropometric indices. The test results were accurate to 0.1 kg and 0.1 cm, respectively. All participants were asked to fast for at least 12 h before blood samples were collected the next morning. Blood samples were collected from the cubital veins to analyze plasma levels of creatinine (CR), fasting glucose (FPG), triglycerides (TG), aspartate aminotransferase (AST), alanine aminotransferase (ALT), low-density lipoprotein cholesterol (LDL-C), and high-density lipoprotein cholesterol (HDL-C). A complete description of the procedures for storing blood samples and the measurement of laboratory indicators can be found elsewhere [13,14].

Echocardiographic examination was performed using a commercially available Doppler echocardiograph (Vivid, GE Healthcare, Chicago, IL, USA) with a 3.0-MHz transducer, including M-mode, 2-dimensional, spectral and color Doppler. Echocardiogram analyses and readings were conducted by three doctors who specialized in echocardiography. Under the guideline of the American Society of Echocardiography [15], the parasternal long-axis view was measured to record aortic annular diameter (AOD), left atrial diameter (LAD), interventricular septal thickness (IVSd), LV end-diastolic internal dimension (LVIDd), LV end-systolic internal dimension (LVIDs), and posterior wall thickness (PWTd). The method of echocardiography has been described previously [15,16]. The LV end-diastolic volume (LVEDV) and LV end-systolic volume (LVESV) were estimated by Teichholz equations: LVEDV (mL)  =  LVIDd3 × 7.0/(2.4 + LVIDd), LVESV (mL)  =  LVIDs3 × 7.0/(2.4 + LVIDs). LV ejection fraction (LVEF) was calculated as [(LVEDV − LVESV)/LVEDV] × 100%, and fractional shortening (FS) was determined with [(LVIDd − LVIDs)/LVIDd] × 100%. We applied pulsed-wave Doppler to record the early diastolic peak flow (E) and atrial peak flow (A) of the mitral valve in the apical four-chamber view.

### 2.3. Definition

Hypertension was defined as systolic blood pressure ≥140 mmHg and/or diastolic blood pressure ≥90 mmHg or the use of anti-hypertensive medications [17]. Body mass index (BMI) was defined as body weight divided by the square of height [18]. Diabetes was diagnosed according to the WHO criteria: fasting plasma glucose ≥7 mmol/l (126 mg/dl) and/or being on treatment for diabetes [19]. The Framingham score was calculated according to the sex-specific equation and included the following risk factors: age (years), total cholesterol (mg/dL), HDL (mg/dL), systolic blood pressure (SBP) (mmHG), diastolic blood pressure (DBP) (mmHG), current smoking (yes/no) and diabetes (yes/no). The ACEF score was calculated according to the following formula: ACEF = age/LVEF + 1 (if the serum creatinine (sCr) level was  >2 mg/dL) [20].

### 2.4. Adjudication of Endpoints

In the present analysis, the endpoint was CVD incidence, defined as stroke or coronary heart disease (CHD) during the follow-up period. Stroke events were defined as rapidly developing signs of focal (or global) disturbance of cerebral function lasting >24 h (unless interrupted by surgery or death) with no apparent nonvascular causes [21]. CHD was defined via a diagnosis of hospitalized angina, hospitalized myocardial infarction, any revascularization procedure, or CHD-related death [22]. When participants reported a possible diagnosis or death of the participant was recorded, all available clinical data, such as medical records, relevant imaging data, and death certificates, were collected. The endpoint assessment committee was responsible for reviewing and adjusting the data independently.

### 2.5. Statistical Analysis

The values of continuous variables are expressed as mean ± standard deviation (SD) or median (Q1–Q3 quartiles) and numbers (percentages) for categorical variables. The differences in clinical characteristics between groups were analyzed by Student’s *t*-test. Results of the Kaplan–Meier survival curve and the log-rank test were applied. Univariate and adjusted Cox proportional hazards models with hazard ratios (HR) and 95% confidence intervals (CI) were performed. The ACEF scores were analyzed as continuous variables (per 1-SD) and categorical variables with quartiles cutoff. The association between ACEF and CVD was also examined using a restricted cubic spline (RCS) model. RCS is used to fit the relationship between the independent and ending variables of the regression model. The horizontal coordinate represents the independent variable of the regression model, and the vertical coordinate indicates the ending variable of the regression model. It enables us to visualize how the ending variable accompanies the independent variable [23]. The predictive value added by ACEF in original models was assessed by the C-statistic, continuous net reclassification improvement (NRI) and integrated discrimination improvement (IDI). This original model involves a collection of major CVD risk factors, including sex, BMI, drinking, smoking, hypertension, diabetes, LDL-C, TCH, and TG. Previous studies have included these indicators in risk prediction models for cardiovascular disease [24,25,26]. Then, we selected these risk factors based on the information collected during our follow-up study. We also evaluated the calibration of the original model by the Hosmer–Lemeshow goodness of fit test (*p* = 0.772). For all analyses, 2-tailed *p* values < 0.05 were considered statistically significant. All statistical analyses were performed with SPSS version 26.0 software (SPSS Inc., Chicago, IL, USA) and R version 4.1.1 (R Foundation for Statistical Computing, Vienna, Austria).

## 3. Results

The baseline and procedural characteristics of 8613 participants are presented in detail in Table 1. The mean age of all participants was 52.9 ± 10.3. Of the total participants, 46.1% were male. Participants characterized with the primary endpoint (CVD) had significantly higher ACEF levels and were older compared to those without CVD. Among patients with CVD, a higher prevalence of smoking and alcohol consumption was observed, along with a higher incidence of hypertension and diabetes. In addition, participants who had CVD showed higher creatinine levels and lower eGFR.

**Figure 2:**
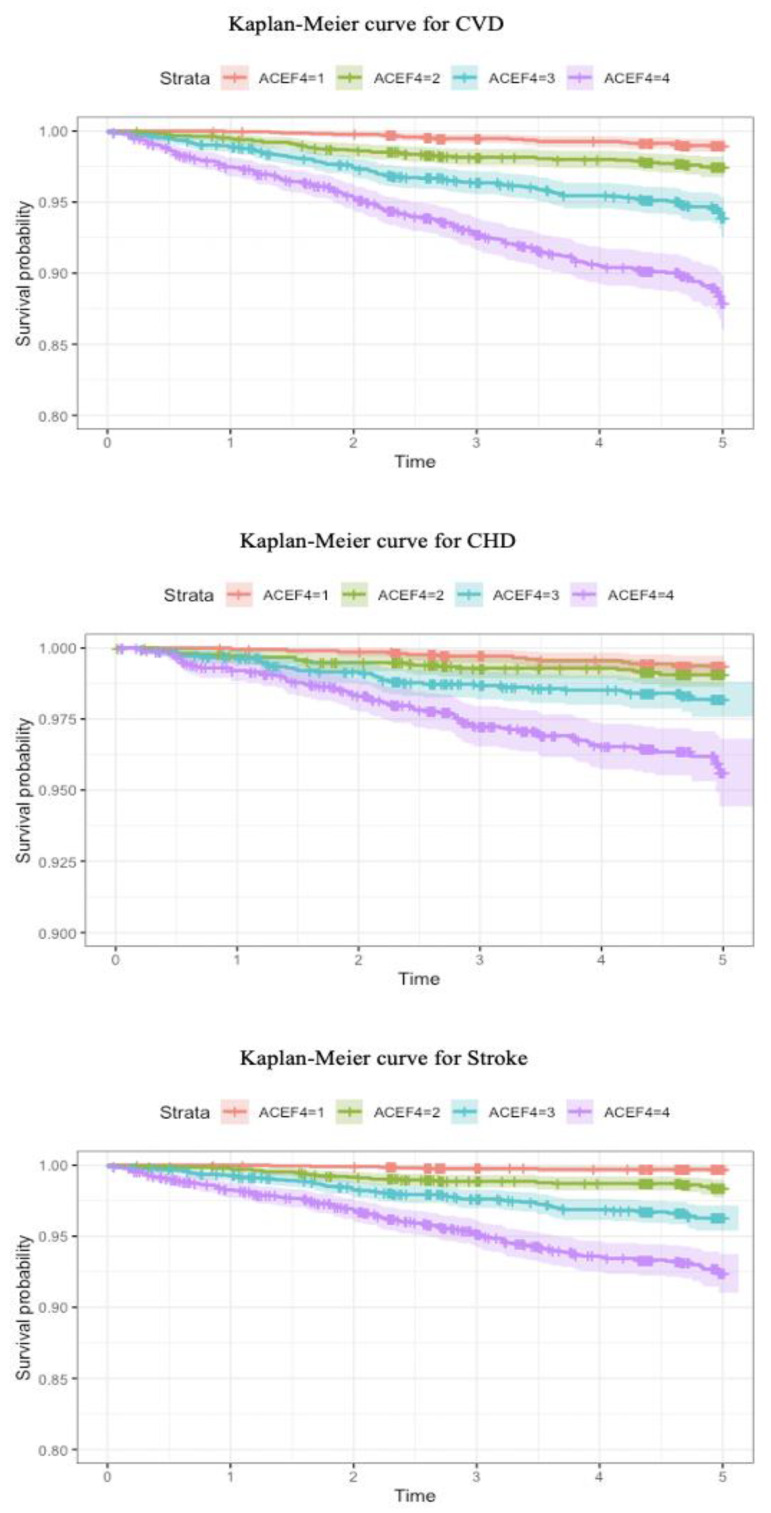
Kaplan–Meier curves for CVD according to different quartiles of ACEF4 = 1: red line represents 1st quartile, ACEF4 = 2: green line represents 2nd quartile, ACEF4 = 3: blue line represents 3rd quartile, and ACEF4 = 4: purple line represents 4th quartile. The time unit of the abscissa is years.

During a mean follow-up period of 4.66 years, 388 CVD events were recorded. We divided all participants by quartiles of ACEF scores (1st quartile: ACEF < 0.706; 2nd quartile: 0.706 ≤ ACEF < 0.823; 3rd quartile: 0.823 ≤ ACEF < 0.964; 4th quartile: ACEF ≥ 0.964). As shown in Figure 2, Kaplan–Meier analysis of the primary endpoint (CVD) demonstrated a significant decrease in survival with increasing ACEF levels. We also evaluated the Kaplan–Meier analysis of secondary endpoints (CHD and stroke). Participants with quartile four of ACEF scores still showed the lowest survival rates with respect to CHD and stroke.

In univariate Cox regression analysis of the primary endpoint (Table 2), participants with high ACEF scores were significantly more likely to be diagnosed with CVD compared to participants with low ACEF scores (1st quartile as a reference; 2nd quartile: HR = 2.59, 95% CI: 1.52–4.39, *p* < 0.001; 3rd quartile: HR = 5.81, 95% CI: 3.57–9.47, *p* < 0.001; 4th quartile: HR = 9.92, 95% CI: 6.10–16.13, *p* < 0.001). After adjusting for traditional cardiovascular risk factors, the risk of CVD still increased in parallel with the ACEF score quartiles (1st quartile as a reference; 2nd quartile: adjusted HR = 2.33, 95% CI: 1.37–3.98, *p* = 0.002; 3rd quartile: adjusted HR = 4.81, 95% CI: 2.93–7.88, *p* < 0.001; 4th quartile: adjusted HR = 8.00, 95% CI: 5.44–11.81, *p* < 0.001). In the COX regression analysis conducted with other secondary endpoints (CHD, stroke), the probability of events remained higher for participants with higher ACEF scores. For CHD and stroke, the adjusted HRs for the fourth quartile were 4.78 (2.54–9.02, *p* < 0.001) and 7.21 (2.69–19.36, *p* < 0.001), respectively. In addition, ACEF scores were independently associated with CVD risk in a continuous analysis after adjustment for various cardiovascular risk factors (ACEF score increased by 1 SD, adjusted HR = 1.95, 95% CI: 1.74–2.20, *p* = 0.026).

**Figure 3:**
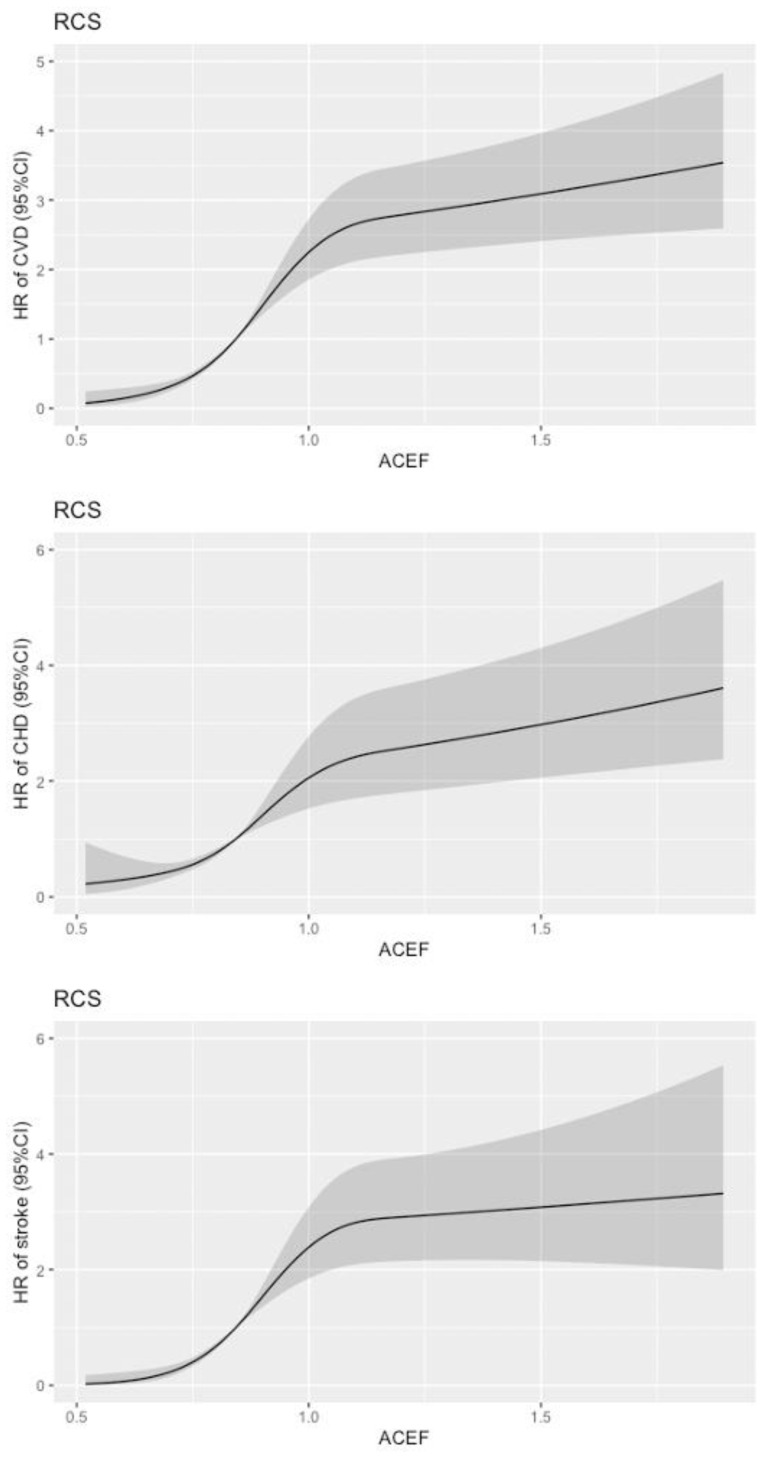
Restricted cubic spline plot of ACEF score and risk of CVD. The vertical coordinate is hazard ratio (HR), and the horizontal axis is ACEF score.

**Figure 4:**
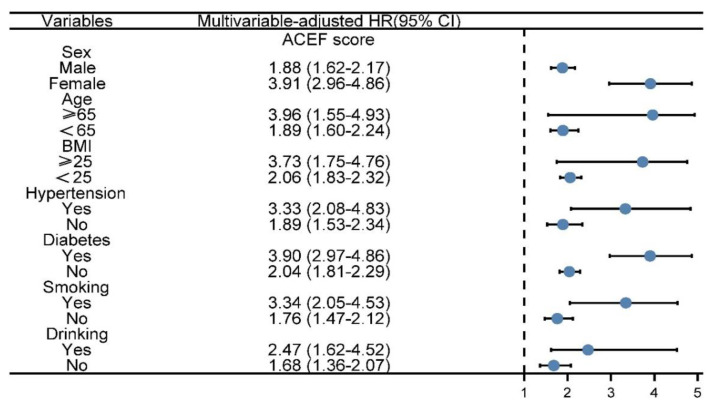
Multivariate adjusted subgroup analysis of ACEF score in relation to CVD. Adjusted for sex, BMI, drinking, smoking, TCH, TG, diabetes, hypertension, LDL-c, TCH, and TG. SD, standard deviation.

The restricted cubic spline (RCS) showed a non-linear relationship between the ACEF and CVD risks on a continuous scale (Figure 3). We observed that the risk of CVD increased rapidly with the ACEF score (0.5–1), followed by a reduction in the increase (>1). The findings indicate a non-linear relationship between ACEF scores and the risk of CVD. Using RCS, we visualized the predicted risk relationship between ACEF score and CVD. RCS also showed a non-linear relationship between ACEF scores and the risk of CHD and stroke. As ACEF increased, the risk of CVD also increased. Meanwhile, we also observed that the associations between ACEF and secondary endpoints were also non-linear. We then performed a subgroup analysis based on clinical characteristics (Figure 4). The multivariate-adjusted HR for ACEF also tended to increase for CVD when stratified by sex, age, BMI, hypertension, diabetes, smoking, and drinking.

Finally, we evaluated whether ACEF could be used to improve CVD risk stratification in the general population and compared ACEF scores with the Framingham score. As shown in Table 3, the C-statistic showed that the addition of ACEF to the original model comprising traditional risk factors resulted in a significant improvement in the predictive power of primary CVD. Compared to the Framingham score, the C-statistic of the risk prediction model with ACEF was higher than the Framingham score (ACEF: 0.690, Framingham: 0.685). In the meanwhile, the increase in NRI and IDI (NRI: 0.543, IDI: 0.0166) further suggested that ACEF can improve risk stratification for CVD.

## 4. Discussion

We present a large cohort study that validates, for the first time, the predictive value of ACEF scores for CVD in the general population and demonstrates that elevated ACEF scores are strongly associated with the risk of CVD, CHD, and stroke. After adjusting for potential confounding variables, participants in the second-fourth quartiles of ACEF scores showed a 2.33–8 fold increased risk of being diagnosed with primary endpoints compared with the participants in the first quartile. In addition, after adding ACEF to the conventional risk factor model, a significant positive effect was observed on the predictive value of the model. Hence, ACEF scores may be reliable as a risk stratification tool in CVD-related clinical studies.

CVD is the leading cause of death in China [27]. Risk prediction in populations is an effective and cost-efficient method for reducing the risk of CVD [28,29]. Risk prediction improves the perception of the patient regarding the risk and prevents further development of CVD. Therefore, a tool that can effectively stratify CVD risk in the general population is very important and necessary. Our findings provide an alternative to this issue.

ACEF was originally used to assess the risk of death from cardiac surgery; however, recent studies have demonstrated the predictive value of ACEF scores in patients with various CVDs. In a cohort study of 1179 patients with myocardial infarction, ACEF scores showed good predictive value for adverse cardiovascular events [30]. Recently, a study conducted to investigate the predictive value of ACEF scores in 1901 patients with acute coronary syndrome found that the scores were associated with an increased risk of all-cause mortality and stroke [31]. In addition, in a controlled trial assessing ACEF scores in patients with myocarditis at risk of serious cardiovascular events, ACEF scores were validated as a significant predictor of serious cardiovascular events [32]. Moreover, ACEF scores are for the risk assessment of patients with myocardial infarction and provide a valid prognostic value [33]. Previous studies have focused on investigating the prognostic role of ACEF scores in patients with various cardiovascular diseases. Our study further investigated the role of ACEF scores in predicting cardiovascular outcomes in the general population in order to elucidate the potential relationship between ACEF scores and cardiovascular risk. We further validated that ACEF can be used as a valid CVD risk stratification tool in a general population cohort.

In our study, the proportion of patients with CVD increased gradually from the first ACEF group to the fourth ACEF group, and participants with high ACEF scores had a higher risk of being diagnosed with both primary and secondary endpoints in the general population. Furthermore, we observed a positive correlation between ACEF and CVD incidence based on RCS curves. We believe that ACEF scores can be a significant predictor of CVD in the general population, which can be attributed to the variables that are used to characterize ACEF. Three variables of ACEF, including age, LVEF, and creatinine, are associated with the risk of CVD. Aging is a well-known independent predictor of CVD diagnosis [34]. In community-dwelling adults without CVD, LVEF in the supra-normal range is associated with a higher risk of adverse cardiovascular outcomes [35]. Chronic kidney disease has been widely recognized as a risk factor for CVD. Creatinine as a kidney marker could improve cardiovascular risk prediction [36,37]. These results provide evidence for the use of ACEF as a tool to predict CVD risk in the general population. In addition, ACEF shows better clinical applicability for avoiding overfitting compared to more complex risk models. The variables included in ACEF scores are readily available and do not require additional calculations.

Our research can help clinicians with patient counseling and monitoring of CVD risk. The ACEF score can accurately predict CVD risk in the general population, which provides a valuable tool for large population-based epidemiological studies. Meanwhile, clinicians should monitor individuals at high-risk for CVD closely to aid with timely interventions.

This study has several limitations. First, only baseline ACEF scores were calculated without assessing changes during follow-up, which could lead to misclassification. Secondly, although multivariate adjustment and subgroup analysis were performed, other residual confounding factors may affect the prognosis. The fact that some participants were excluded could cause bias in the results of the study. Finally, we could not collect therapeutic data for the population, which may lead to a certain bias in our results.

In conclusion, our study showed, for the first time, that ACEF scores could be independent predictors of CVD, CHD, and stroke in the general population. ACEF scores may help identify high-risk patients for further CVD risk stratification and help analyze important clinical implications for CVD prevention in the general population.

## Figures and Tables

**Table 1:** Baseline characteristics of study population.

Variables	Total (*n* = 8613)	CVD (*n* = 388)	Non-CVD (*n* = 8225)	*p* Value
Age, year	52.9 ± 10.3	61.1 ± 9.6	52.5 ± 10.1	<0.001
Male sex, *n* (%)	3974 (46.1)	202 (52.1)	3772 (45.9)	0.004
Body mass index, kg/m^2^	24.7 ± 3.6	25.3 ± 3.6	24.7 ± 3.6	0.009
Currently smoking, *n* (%)	3068 (35.6)	157 (40.5)	2911 (35.4)	0.115
Currently drinking, *n* (%)	2053 (23.8)	104 (26.8)	1949 (23.7)	0.035
Hypertension, *n* (%)	4123 (47.9)	282 (72.7)	3841 (46.7)	<0.001
Diabetes, *n* (%)	765 (8.9)	61 (15.7)	704 (8.6)	0.005
LVEF (%)	62.9 ± 3.8	61.7 ± 4.6	62.9 ± 3.8	<0.001
TG, mmol/L	1.6 ± 1.4	1.7 ± 1.5	1.5 ± 1.4	0.157
TCH, mmol/L	5.2 ± 1.1	5.5 ± 1.1	5.2 ± 1.1	<0.001
LDL-C, mmol/L	2.9 ± 0.8	3.1 ± 0.9	2.9 ± 0.8	<0.001
HDL-C, mmol/L	1.4 ± 0.4	1.4 ± 0.4	1.4 ± 0.4	0.415
ALT, IU/L	22.5 ± 18.8	23.2 ± 20.5	22.4 ± 18.7	0.793
AST, IU/L	22.2 ± 12.1	24.3 ± 15.9	22.1 ± 11.9	0.009
eGFR, ml/min	94.5 ± 14.9	87.1 ± 14.7	94.8 ± 14.8	<0.001
FPG, mol/L	5.8 ± 1.5	6.1 ± 1.6	5.8 ± 1.5	0.009
CR, mmol/L	70.9 ± 18.3	75.1 ± 40.7	70.7 ± 16.5	<0.001
ACEF	0.84 ± 0.2	1.0 ± 0.2	0.84 ± 0.2	<0.001

Data are expressed as the mean value ± standard deviation, median with 25th and 75th or number (%). Abbreviations: ALT, alanine aminotransferase; AST, aspartate aminotransferase; HDL-C, high-density lipoprotein cholesterol; LDL-C, low-density lipoprotein cholesterol; TCH, total cholesterol; TG, triglycerides; PLT, platelet; FPG, fasting plasma glucose; eGFR, estimated glomerular filtration rate.

**Table 2:** Univariate and multivariate Cox proportional hazards regression analysis of CVD events.

Event	ACEF	Univariate Model		Adjusted Model	
		HR (95% CI)	*p* Value	HR (95% CI)	*p* Value
CVD					
	Per 1 SD increment	2.11 (1.90–2.33)	<0.001	1.95 (1.74–2.20)	0.026
	<0.706 (1st quartile)	1.00 (reference)		1.00 (reference)	
	0.706–0.823 (2nd quartile)	2.59 (1.52–4.39)	<0.001	2.33 (1.37–3.98)	0.002
	0.823–0.964 (3rd quartile)	5.81 (3.57–9.47)	<0.001	4.81 (2.93–7.88)	<0.001
	>0.964 (4th quartile)	9.92 (6.10–16.13)	<0.001	8.00 (5.44–11.81)	<0.001
CHD					
	Per 1 SD increment	2.12 (1.79–2.49)	<0.001	1.80 (1.16–2.78)	0.008
	<0.706 (1st quartile)	1.00 (reference)		1.00 (reference)	
	0.706–0.823 (2nd quartile)	1.58 (0.76–3.25)	0.21	1.39 (0.67–2.88)	0.37
	0.823–0.964 (3rd quartile)	3.09 (1.62–5.93)	0.001	2.38 (1.22–4.65)	0.011
	>0.964 (4th quartile)	6.42 (3.49–11.81)	<0.001	4.78 (2.54–9.02)	<0.001
Stroke					
	Per 1 SD increment	2.10 (1.85–2.39)	<0.001	1.96 (1.70–2.25)	<0.001
	<0.706 (1st quartile)	1.00 (reference)		1.00 (reference)	
	0.706–0.823 (2nd quartile)	3.45 (1.49–8.00)	0.004	2.71 (1.15–6.41)	0.023
	0.823–0.964 (3rd quartile)	8.95 (4.10–19.55)	<0.001	5.28 (2.23–12.49)	<0.001
	>0.964 (4th quartile)	17.79 (8.30–28.14)	<0.001	7.21 (2.69–19.36)	<0.001

Adjusted for sex, BMI, smoking, drinking, diabetes, hypertension, LDL, TCH, TG.

**Table 3:** Discrimination and reclassification for CVD with the addition of ACEF to traditional risk factors model.

Model	C-Statistic (95% CI)	NRI (95% CI)	*p*-Value	IDI (95% CI)	*p*-Value
Original model For CVD	0.666 (0.640–0.692)				
Original model + ACEF for CVD	0.692 (0.661–0.723)	0.543 (0.445–0.642)	<0.001	0.0166 (0.0116–0.0216)	<0.001
Framingham score for CVD	0.685 (0.660–0.715)				
Original model For CHD	0.644 (0.600–0.688)				
Original model + ACEF for CHD	0.694 (0.664–0.725)	0.575 (0.419–0.731)	<0.001	0.0062 (0.0024–0.01)	<0.001
Framingham score for CHD	0.682 (0.641–0.722)				
Original model For Stroke	0.676 (0.645–0.708)				
Original model + ACEF for Stroke	0.690 (0.659–0.720)	0.618 (0.460–0.677)	<0.001	0.0077 (0.0055–0.01)	<0.001
Framingham score for Stroke	0.681 (0.666–0.726)				

Original model: sex, BMI, drinking, smoking, hypertension, diabetes, LDL-C, TCH, TG.

## Data Availability

The data that support the findings of this study are available from the corresponding author upon reasonable request (guoxiaofan1986@hotmail.com).
